# Kinetics of Humoral Immunity against SARS-CoV-2 in Healthcare Workers after the Third Dose of BNT162b2 mRNA Vaccine

**DOI:** 10.3390/vaccines10111948

**Published:** 2022-11-17

**Authors:** Tiziana Grassi, Giambattista Lobreglio, Alessandra Panico, Chiara Rosato, Antonella Zizza, Roberta Lazzari, Michele Chicone, Floriano Indino, Francesco Bagordo

**Affiliations:** 1 Department of Biological and Environmental Science and Technology, University of Salento, 73100 Lecce, Italy; 2 Clinical Pathology and Microbiology Unit, Vito Fazzi General Hospital, 73100 Lecce, Italy; 3 Institute of Clinical Physiology, National Research Council, 73100 Lecce, Italy; 4 Department of Pharmacy—Pharmaceutical Sciences, University of Bari Aldo Moro, 70121 Bari, Italy

**Keywords:** SARS-CoV-2, BNT162b2 mRNA vaccine, healthcare workers, booster dose

## Abstract

Protection provided by COVID-19 vaccines is compromised due to waning immunity over time. This study aimed to assess the level of antibodies anti-S-RBD of SARS-CoV-2 in a cohort of healthcare workers before and, on average, one and four months after the third dose of the BNT162b2 vaccine. The determination of antibodies was carried out in serum samples using an electrochemiluminescence immunoassay (ECLIA). All 34 participants (10 males, 24 females, 19 participants <50 years old, 15 participants ≥50 years old) showed a significant antibody level increase after the booster dose. Subsequently, a significant decrease in the antibody concentration was observed, with a reduction of about 60% after 150 days from the booster. Six subjects were infected by SARS-CoV-2 after the booster and showed a significantly higher antibody concentration on average four months after the third dose compared to naïve ones. Male and female participants had a similar trend in the antibody decline, while older subjects, compared to the younger ones, had a slightly slower decrease, even if they developed a lower level of antibodies after the third dose. These findings support the importance of the booster dose and underline the need for surveillance programs to better understand the antibody kinetics and optimize vaccination strategies.

## 1. Introduction

The Coronavirus Disease 2019 (COVID-19) pandemic, caused by the Severe Acute Respiratory Syndrome Coronavirus 2 (SARS-CoV-2), is still ongoing for more than two years. The introduction of effective vaccines against SARS-CoV-2 reduced the viral transmission and disease burden, limiting cases of infection and providing protection against severe clinical forms, hospitalizations, and mortality [1]. 

The efficacy of a vaccine can be assessed by the evaluation of humoral response through the measurement of circulating antibody levels, which represents a reliable immunological correlate of protection for assessing the individual level of protection against infection [2]. In particular, IgG antibodies recognizing the receptor-binding domain (RBD) of the spike protein (anti-S-RBD), which mediates virus entry into target cells, have neutralizing functions and may be used as a correlate of protection [3]. 

Several studies provided data on effectiveness, immunogenicity, and antibody kinetics both after natural infection [4,5] and vaccination [6,7]. Growing evidence suggests that there are differences in the level and duration of protection of natural infection versus vaccination [8,9]. Compared to antibody concentration induced by natural infection, vaccination may induce similar or lower neutralizing antibody levels [10] that decay faster [11]. Nevertheless, a complete picture of the immunity duration is still unclear. 

Vaccination against SARS-CoV-2 elicited an excellent immune response; however, a decay of the antibody level was observed over time. The antibody kinetics six to eight months after primary two-dose vaccination was reported for different vaccines, i.e., BNT162b2 [12], mRNA-1273 [13], and Ad26.COV2.S [14]. Several studies found a decrease in the IgG titer after a few months from the second dose [12,15,16,17]. 

The decline in circulating antibodies has raised concerns and the need to improve the protection against SARS-CoV-2 infection by administering a third dose of vaccine (booster dose). Booster vaccination has been shown to be effective against several different SARS-CoV-2 variants, including the new Omicron variant [18,19].

Understanding the duration of protection gained by a booster dose is critical for guiding vaccine strategies, with a significant impact on public health policy to mitigate the pandemic.

In this study, the level of antibodies anti-S-RBD in healthcare workers was assessed before and, on average, one and four months after the third dose of the BNT162b2 vaccine. The antibody concentration was analyzed according to gender, age, time of serological tests, and SARS-CoV-2 infection.

## 2. Materials and Methods

### 2.1. Study Design and Participants

This study was part of the “COVID-19 Research Project” promoted by the Local Health Authority (ASL) of Lecce and the University of Salento. A group of 38 healthy twice vaccinated healthcare workers naïve to SARS-CoV-2 infection was included in the study. Participants were screened for antibodies anti-S-RBD of SARS-CoV-2 at the Clinical Pathology and Microbiology Unit of the “Vito Fazzi” Hospital in Lecce (Puglia, Italy). 

Inclusion criteria for participation in the study were: complete BNT162b2 vaccination cycle; free from chronic diseases (cancer, autoimmune disorders, etc.); non-clinically, radiologically, or laboratory tests detecting SARS-CoV-2 infection prior to time of enrollment.

The tests were performed before (test A) (about 10–11 months after the second dose) and about one (test B) and four months (test C) after the third dose of the BNT162b2 vaccine. At the same time, for every test, the cohort was also screened for antibodies anti-nucleoprotein (N) of SARS-CoV-2 in order to reveal a previous SARS-CoV-2 infection. The data of participants who tested positive for antibodies anti-N were analyzed separately. The cohort was previously investigated, and preliminary results were reported [6]; during the follow-up, four subjects were lost.

### 2.2. Data Collection

For each subject, the age, sex, vaccination status (number of doses, dates, and type of vaccine), dates of blood draws for serological tests, and data on positivity to COVID-19 were recorded. A signed informed consent form was obtained from all subjects for research data collection.

### 2.3. Detection of Antibodies Anti-(S) RBD

The determination of antibodies anti-(S) RBD of SARS-CoV-2 was carried out in serum samples using the Elecsys^®^ Anti-SARS-CoV-2 S (Roche Diagnostics GmbH, Mannheim, Germany), an electrochemiluminescence immunoassay (ECLIA) developed for the in vitro quantitative detection of total antibodies (including immunoglobulin G) against the SARS-CoV-2 S protein RBD in human serum and plasma. The assay uses a recombinant protein representing the RBD of the S protein in a double-antigen sandwich assay format, which favors the detection of high-affinity antibodies against SARS-CoV-2. According to the manufacturer’s instructions, the measuring range spanned from 0.4 to 250 U/mL (up to 25,000 U/mL with onboard 1:100 dilution on a Cobas 8000 analyzer); values higher than 0.8 U/mL were considered positive.

According to the manufacturer, the sensibility and specificity of the test were 98.8% and 99.9%, respectively.

### 2.4. Detection of Antibodies Anti-Nucleoprotein (N)

The antibodies anti-S are produced as a consequence of both natural infection and vaccination by the BNT162b2 vaccine that induces the production of the antigen S. In order to discriminate these conditions, the presence of antibodies anti-N was analyzed since it is directly linked to the SARS-CoV-2 infection but not to the vaccination. In this study, the Elecsys^®^ Anti-SARS-CoV-2 N (Roche Diagnostics GmbH, Mannheim, Germany), which uses a recombinant protein representing the N antigen in a double-antigen sandwich assay format, was used to identify subjects with a history of previous SARS-CoV-2 infection and to exclude immunization due to infection rather than vaccination. This test showed 97.2% sensitivity and 99.8% specificity for detecting preceding SARS-CoV-2 infection [20].

### 2.5. Statistical Analysis

Data on subjects’ details and serological analysis were entered in a Microsoft Excel database and statistically analyzed using MedCalc Software version 12.3 (MedCalc Software bvba, Ostend, Belgium).

The general characteristics of the study population were summarized by means of a descriptive statistic. In each group, the quantitative variables were reported as mean ± standard deviation (SD) while the qualitative variables as frequencies (%). The distribution of antibody concentrations was represented graphically through a boxplot in which the results were grouped into interquartiles, and the minimum, first quartile, median, third quartile, and maximum, as well as any outliers, were highlighted. Any differences in the humoral responses between the groups were evaluated by the non-parametric Kruskal–Wallis test since the small sample size did not allow for verifying a normal distribution of the values.

Finally, a polynomial (second-degree) regression model was applied to describe the mean trend in antibody concentration over time before and after the booster dose of the vaccine. In addition, a simple linear regression was performed to highlight the antibody level trend over time as a function of the gender and age of the participants after the booster dose. The Student *t*-test was performed to determine whether the slopes of the regression lines were significantly different from each other, and any difference was considered significant if *p*-value < 0.05.

### 2.6. Ethical Aspects

The study was approved by the Ethical Committee of the Lecce Local Health Authority (ASL/LE) on 29 May 2020 with deliberation n. 557. All data were collected and analyzed confidentially in accordance with Italian laws (Legislative Decree n. 196 of 30 June 2003, and subsequent additions) for research purposes.

## 3. Results

The general characteristics of the study population are reported in Table 1. Of the 38 healthcare workers recruited at the start of the study, a total of 34 of them, 10 (29.4%) males and 24 (70.6%) females, completed the full test cycle (one test before and two tests after the booster dose). The average age was 47.0 ± 11.5 years, 15 (44.1%) subjects were ≥ 50 years old, and 19 (55.9%) were <50 years old. All participants received three doses of BNT162b2 vaccine, and all tested positive for antibodies anti-(S)RBD before and after the booster dose. The mean time interval between test A and the third dose was 20 ± 16 days, between the third dose and test B was 34 ± 16 days, and test C was performed on average 124 ± 12 days after the booster dose. 

Six subjects (17.6%) had COVID-19 after the third dose of vaccine, between tests B and C, and resulted positive for antibodies anti-N at test C. These subjects were previously tested as negative for anti-N antibodies; therefore, any difference in the antibody anti-S level can be attributed to inter-individual variability in the vaccination response. All were pauci-symptomatic, and only one developed mild symptoms (COVID#4). Test C was performed on average 59 ± 12 days after the positivity (mean ± SD of difference between columns 9 and 8 in Table 2). Data on their antibody titers, time interval between the third dose, tests, and positivity for SARS-CoV-2 are reported in Table 2.

**Figure 1:**
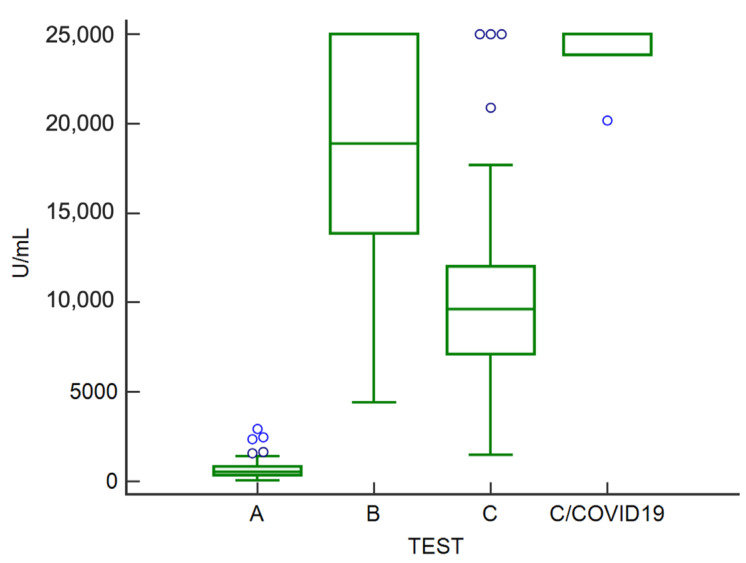
Box-plot showing the distribution of antibody concentration in analyzed subjects before (test A) and on average one (test B) and four months (test C for naïve subjects, test C/COVID19 for infected subjects) after the third dose. Blue circles represent the outliers. (Test A: prior booster; Test B: one month after booster; Test C: four months after booster).

In general, the mean antibody titer was 763.1 ± 687.8 U/mL at test A, 17,679.0 ± 6420.0 U/mL at test B, and, as for subjects free of infection, 10,919.2 ± 6528.6 U/mL at test C. Participants who resulted positive for SARS-CoV-2 showed a higher level of antibodies (Figure 1), with a mean of 24,004.5 ± 1938.3 U/mL at test C. The mean value of antibody titer was significantly different (*p* < 0.001) among all groups.

Starting from a three-point survey for each subject and considering that the tests were conducted in a more or less wide time interval (Table 1), we built population regression models that extend up to the 150th day from the booster dose. 

**Figure 2:**
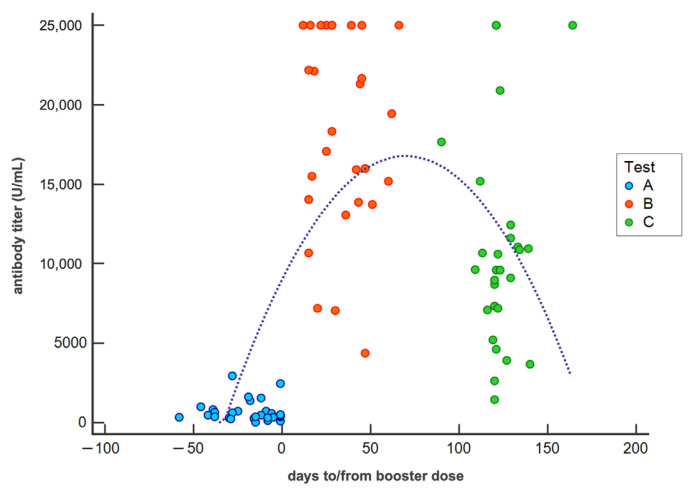
Scatter diagram showing the antibody titer (detection limits 0.4–25,000 U/mL) of each naïve subject measured in tests A, B, and C against time and related regression curve. (Test A: prior booster; Test B: one month after booster; Test C: four months after booster).

Figure 2 represents the temporal distribution of all antibody titers measured in the population included in the study and describes the antibody kinetics measured before (test A) and after the booster dose (test B and test C) in COVID-19-free subjects. The regression equation (y = 9057.3529 + 221.6632x + −1.5899 × 2, R^2^ = 0.3895) describes an increase in the antibody titer after the booster dose with a maximum value on about the 60th days equal to a concentration of approximately 17-fold higher than the measurement performed before the booster dose. Subsequently, a rapid decrease in the antibody concentration was observed, with a reduction of about 60% after 150 days from booster dose.

**Figure 3:**
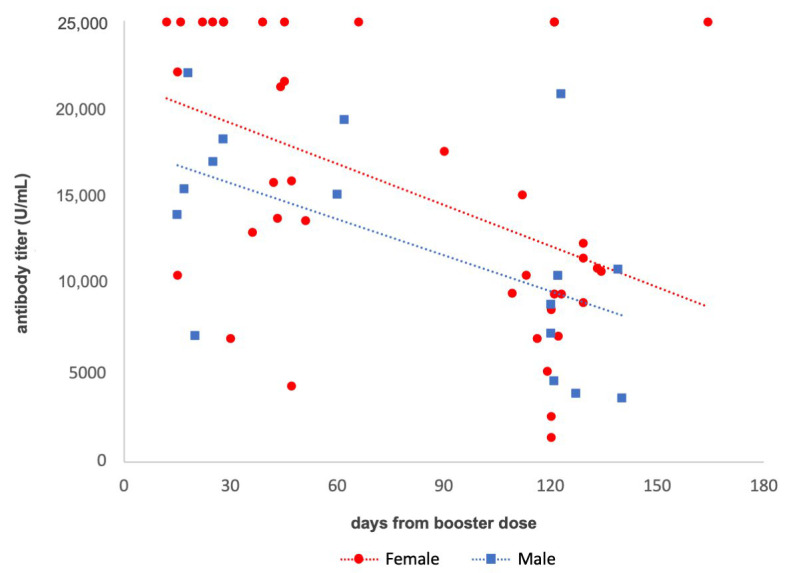
Scatter diagram showing the antibody titer (detection limits 0.4–25,000 U/mL) after the booster dose of female and male subjects against time and related regression lines.

The antibody kinetics in naïve participants determined at test B and C and analyzed by gender (red for females and blue for males) is shown in Figure 3. No significant differences (*p* > 0.05) between groups were observed according to age (male 45.1 ± 12.6 years; female 45.8 ± 11.6 years) and interval between vaccinations (male 281.9 ± 5.1 days; female 278.2 ± 49.4 days). The antibody titers of each subject were entered in a dispersion diagram, in which the antibody titer measured in each subject is shown in the ordinate and the time (days) in which the test was carried out with respect to the booster (time 0) in the abscissa. The maximum detectable titer was 25,000 U/mL; therefore, several dots are aligned at the top of the graph. A regression line was calculated as a function of the time (days) from the booster (female: y = −77.539x + 21,579, R^2^ = 0.2176; male: y = −68.03x + 17,838, R^2^ = 0.3233). The results highlighted that women had a slightly more pronounced antibody response than men. Additionally, the antibody concentration waned progressively over time without significant differences between sexes (*p* > 0.05). Indeed, the mean antibody concentration measured for women 30 days after the booster dose was 19,253 U/mL, 12,274 U/mL after 120 days (with a decrease of 36.0%), and 9948 U/mL after 150 days (with a decrease of 48.0%). Male subjects showed a mean antibody titer of 15,797 U/mL 30 days after the booster dose, 9674 U/mL after 120 days (with a decrease of 38.8%), and 7634 U/mL after 150 days (with a decrease of 51.7%).

According to age, participants were divided into two groups: <50 and ≥50 years old. No significant differences (*p* > 0.05) among groups were observed in relation to time interval between vaccinations (<50: 283.0 ± 45.8 days; ≥50: 274.8 ± 37.5 days). In addition, the age distribution according to gender between the two groups was similar (*p* > 0.05) (<50: female 35.6 ± 5.8 years; male 36.4 ± 5.8 years) (≥50: female 55.9 ± 4.9 years; male 59.7 ± 2.9 years).

**Figure 4:**
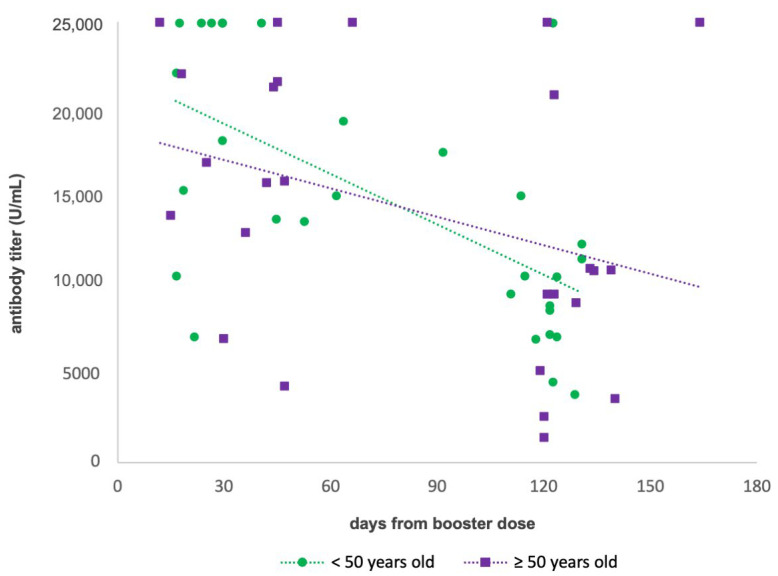
Scatter diagram showing the antibody titer (detection limits 0.4–25,000 U/mL) after the booster dose of <50 years old and ≥50 years old subjects against time and related regression lines.

A scatter diagram (Figure 4) was performed, and a regression line was calculated for subjects <50 years old and ≥50, respectively (<50: y = −95.307x + 22,008, R^2^ = 0.3861; ≥50: y = −53.832x + 18,804, R^2^ = 0.1153). The slopes of two lines were not significantly different (*p* > 0.05). However, participants <50 years old seemed to have a slightly more rapid decline in the antibody titer than the older ones. In particular, subjects <50 years old showed a mean antibody concentration of 19,149 U/mL 30 days after the booster dose, 10,571 U/mL after 120 days (with a decrease of 44.8%), and 7712 U/mL after 150 days (with a decrease of 59.7%). People ≥50 years old showed a mean antibody titer of 17,189 U/mL after 30 days from the third dose, 12,344 U/mL after 120 days (with a decrease of 28.2%), 10,729 U/mL after 150 days (with decrease of 37.6%).

## 4. Discussion

It is now universally recognized that vaccination is the safest and optimal tool to protect people against COVID-19 infection, hospitalization, and death [1].

The evaluation of antibody concentration can be considered a reliable, easy-to-perform, and low-cost tool for assessing vaccine efficacy. A robust correlation between higher antibody titers and vaccine efficacy was recently described [21,22]. Another study found that RBD-specific IgG correlated with neutralizing antibody titers and RBD-specific memory B cell frequencies [23]. In addition, a high correlation between IgG against the RBD and neutralizing antibody titers was observed in a recent survey [24], suggesting that IgG could serve as a correlate of neutralization. However, correlates of protection have not yet been determined. 

The antibody level does not remain constant over time, and a decrease was observed about five to six months after the second dose [12,16,17]. In order to overcome the waning of immunity, an additional vaccine dose was administered, firstly for frail people and workers of essential public activities and secondly for the general population.

In this study, the level of antibody anti-S-RBD of SARS-CoV-2 was assessed in healthcare workers before and after the booster dose of the BNT162b2 vaccine. The BNT162b2 vaccine elicits an excellent immune response, with a rapid increase in antibody level to approximately 17-fold higher than the level measured before the booster dose around the 60th day after the vaccine administration. A gradual decrease in the antibody titer was subsequently registered, with a reduction of about 60% five months after the third dose.

Overall, previous research on healthy individuals investigated the antibody kinetics 4–6 months after the second vaccine dose describing a significant reduction of antibody concentration. Brisotto et al. [25] showed a significant antibody decrease independent of age and sex in a cohort of 767 healthcare workers four months after two-dose vaccination. Stamatopoulou et al. [26] found that the anti-SARS-CoV-2 IgG levels decreased significantly four months after the second dose in a cohort of 142 infection-naïve healthcare workers. Similarly, other studies [27,28] showed a marked waning in anti-RBD antibody levels at six months in vaccinated healthcare workers. 

A similar trend can be observed after the third dose of the vaccine, with a rapid increase after the booster administration and a subsequent decline, as shown by our results. Recently, some Authors assessed the humoral response induced by the booster dose. Eliakim-Raz et al. showed that a third BNT162b2 dose in adults ≥60 years old was associated with a significant antibody level increase [29]. Moreover, Gilboa et al. demonstrated a rapid and robust immune response to the third BNT162b2 dose, characterized by a significant increase in anti-RBD IgG levels [30]. Another study showed a rapid decline of RBD-IgG levels after the second dose in nursing home residents and a significant increase after the administration of the third dose [31]. Lo Sasso et al. [7] investigated the kinetic of anti-S-RBD IgG antibody levels in a cohort of vaccinated subjects with two and three doses, and they observed that antibody levels gradually decreased to a steady state after four months since the peak. Moreover, this decline was found to be independent of age, sex, vaccine doses, and baseline antibodies titer. 

The effectiveness of the third dose is demonstrated not only from a serological point of view but also by the clinical evidence. Indeed, a study conducted in Israel showed a significant reduction of confirmed SARS-CoV-2 infections and severe illness among subjects ≥60 years old who received the booster dose [32]. On the other hand, the retrospective study of Patalon et al. [33] showed that the BNT162b2 third dose effectiveness significantly decreased each month since vaccination. In particular, the protection against infection waned from 53.4% one month after vaccination to 16.5% three months after vaccination.

Recent studies investigated the duration of the humoral response after the SARS-CoV-2 infection showing the persistence of IgG some months after the infection, which is strongly correlated with neutralizing antibody titer [34,35]. Our study showed that the level of antibody anti-S-RBD in subjects who received three doses of vaccine and were subsequently infected with SARS-CoV-2 was significantly higher than those of the naïve participants. 

Some works suggested that antibody titers in previously infected subjects are significantly higher than in SARS-CoV-2 naïve vaccinated individuals [22,36]. 

Sariol et al. [37] evaluated the immune response in a vaccinated cohort comprising both pre-exposed individuals to the infection and naïve ones. They showed that antibody titers remained detectable at high levels for four to seven months after natural infection. Moreover, the quantity and the quality of the antibody response induced by the natural infection resulted in significantly higher titer of binding and neutralizing antibodies when compared to the response induced by mRNA vaccination. For both groups, a rapid decline of antibodies was observed 40 to 80 days (for naïve and pre-exposed subjects, respectively) after the second dose; this decay was more precipitous in the naïve group than the pre-exposed group. Antibodies generated after the natural infection were significantly better in their function. According to the Authors, these results suggested that natural infection with SARS-CoV-2 may contribute to the expansion of memory B cells, enabling the production of more S-specific antibodies following vaccination.

Another study [5] found minimal differences between natural infection protection and vaccine protection immediately after the second vaccine dose, while a divergence between the two types of protection occurred in subsequent months, with a very slow waning in natural infection immunity (at least against pre-Omicron variants) than vaccine immunity. Our results, even only from a few infected subjects, are in line with these findings. However, more longitudinal studies should be performed in order to better define the duration and kinetics of antibodies in vaccinated and then-infected subjects.

In addition, it is particularly noteworthy that several of the COVID-19 subjects in our cohort had high antibody titers at test B. This suggests that high levels of IgG may not necessarily correlate with neutralization and/or protection against infection but could be protective against severe forms of the disease [38] since all of these subjects did not show severe symptoms.

In the present study, the influence of age and gender on antibody kinetics was also investigated. The decrease of the antibody level after the booster dose resulted independent of age and gender. Indeed, as for gender, females had, on average, a slightly more pronounced humoral response to the third dose than males. Subsequently, the decrease in antibody levels between the two sexes appeared to be similar, as evidenced by the slope of the regression lines. Regarding age, younger participants, after the third dose, showed a slightly higher humoral response than participants aged 50 or over due to a general lower effectiveness of the immune system and a lower magnitude of memory B cell responses with increased age [39]. Previous studies confirmed that the antibody concentration reached after the second dose was age-dependent, with a significantly higher level in young people than in older ones [6,40], and also gender-dependent, with a lower IgG level in male subjects [40,41]. Regarding the trend of the antibody titer over time, our study highlighted that the decrease in antibody titer in older people seemed slightly faster than in younger ones. Shrotri et al. [42] showed that the reduction of the antibody titer after the second dose was less rapid in subjects ≥65 years, and in particular in vulnerable people, compared to the younger ones. This could be a possible side effect of prior Coronavirus antigen exposure yielding some level of cross-protective immunity. Other surveys stated an age- and gender-independent decrease in antibody levels after the third dose [6,7,43].

The limitations of this study should be mentioned. The evaluation of the humoral response was performed on a small sample of subjects; however, only a few studies investigated the antibody kinetics some months after the booster dose, and our findings may contribute to the understanding of the immunological response to vaccination and to SARS-CoV-2. Another limitation of our work is the lack of cellular immunity testing and neutralizing antibody testing. Indeed, other than the humoral response, the immune response also includes the innate and the cellular-mediated immune responses [44,45]. In particular, the cellular-mediated immune response is proven to be highly effective and durable after the COVID-19 vaccination and against the SARS-CoV-2 infection, including the variants [46,47,48]. Our further investigations will be made in this direction in order to assess the contribution of T-cell-mediated immunity to vaccination efficacy. Moreover, the sera samples collected in this study could be tested against multiple strains of the virus (i.e., WT, delta, omicron) in order to verify the reactivity of antibodies against natural infection due to several variants.

The literature evidence supports the importance of the booster dose, and ongoing surveillance programs are required to assess the continuity of our findings over time.

## 5. Conclusions

In this study, we found that all participants showed a significant antibody level increase after the booster dose of the BNT162b2 vaccine. Subsequently, a significant decrease in the antibody concentration was observed, with a reduction of about 60% after 150 days from the booster dose. Those subjects who were infected by SARS-CoV-2 after the booster showed a significantly higher antibody concentration on average four months after the third dose compared to naïve ones. Male and female participants seemed to have a similar trend in the antibody decline after the booster administration, while older subjects had a slightly slower decrease than the younger ones.

This work provides new and additional insight into understanding the antibody kinetics after vaccination in naïve and COVID-19 subjects, which may be helpful for answering important questions about immunity against SARS-CoV-2 and optimizing vaccine programs.

## Figures and Tables

**Table 1:** Characteristics of the study cohort, antibody titers, and time interval between booster dose and serological tests. (Test A: prior booster; Test B: one month after booster; Test C: four months after booster).

Variables		
Gender Male Female	10 (29.4) 24 (70.6)	N (%) N (%)
Age ≥50 <50	47.0 ± 11.5 15 (44.1) 19 (55.9)	Mean ± SD (years) N (%) N (%)
Antibody titer at test A	763.1 ± 687.8	Mean ± SD (U/mL)
Antibody titer at test B	17679.0 ± 6420.0	Mean ± SD (U/mL)
Antibody titer at test C	10919.2 ± 6528.6	Mean ± SD (U/mL)
Antibody titer at test C in COVID-19 subjects	24004.5 ± 1938.3	Mean ± SD (U/mL)
Interval test A-3rd dose	20 ± 16	Mean ± SD (days)
Interval 3rd dose-test B	34 ± 16	Mean ± SD (days)
Interval 3rd dose-test C	124 ± 12	Mean ± SD (days)

**Table 2:** Characteristics of the subjects resulted positive for SARS-CoV-2, antibody titers, time interval between booster dose, serological tests, and positivity. (Test A: prior booster; Test B: one month after booster; Test C: four months after booster).

COVID-19 Subjects	Age	Gender	Interval Test A-3rd Dose (days)	Antibody Titer Test A (U/mL)	Interval 3rd Dose-Test B (days)	Antibody Titer Test B (U/mL)	Interval 3rd Dose-COVID-19 Positivity (days)	Interval 3rd Dose-Test C (days)	Antibody Titer Test C (U/mL)
COVID#1	61	M	8	750	65	6651	75	147	23,870
COVID#2	55	M	35	508	14	25,000	74	138	25,000
COVID#3	55	F	42	490	26	13,770	70	127	25,000
COVID#4	59	F	42	2362	18	13,871	89	126	20,157
COVID#5	42	F	5	567	49	25,000	61	121	25,000
COVID#6	51	F	11	808	46	25,000	56	120	25,000

## Data Availability

The data presented in this study are available on request from the corresponding authors.
